# Point-of-care assessment of complement activation in the ICU: a feasibility study of rapid terminal complement complex detection in critical illness

**DOI:** 10.3389/fimmu.2026.1860988

**Published:** 2026-08-11

**Authors:** Tam Cung, Søren Erik Pischke, Camilla Schjalm, Bettina Mülleder, Loek Willems, Max Sonnleitner, Erik J.M. Toonen, Tom Eirik Mollnes, Andreas Barratt-Due

**Affiliations:** 1 Department of Immunology, Oslo University Hospital and University of Oslo, Oslo, Norway; 2 Department of Research and Development, Division of Emergencies and Critical Care, Oslo University Hospital, Oslo, Norway; 3 Department of Anesthesia and Intensive Care Medicine, Division of Emergencies and Critical Care, Oslo University Hospital, Oslo, Norway; 4 Institute of Clinical Medicine, Faculty of Medicine, University of Oslo, Oslo, Norway; 5 GENSPEED Biotech GmbH, Rainbach im Mühlkreis, Austria; 6 Research and Development Department, Hycult Biotechnology, Uden, Netherlands; 7 Research Laboratory, Nordland Hospital Trust, Bodø, Norway

**Keywords:** C3, C3d, critical illness, point-of-care measurement, TCC, terminal complement complex, intensive care, complement system activation

## Abstract

**Background:**

Complement activation is increasingly recognized as a key driver in various critical diseases and a potential therapeutic target. However, conventional enzyme-linked immunosorbent assays (ELISAs) for complement protein detection are laboratory-bound and unsuitable for rapid clinical decision-making in the emergency and intensive care unit setting. We evaluated the feasibility of a point-of-care (POC) multiplex micro-ELISA for complement component C3, activation marker C3d and the soluble terminal complement complex (TCC) in a heterogeneous intensive care unit (ICU) population.

**Method:**

Thirty-eight critically ill and three cardiopulmonary bypass patients were prospectively enrolled. Fifteen age- and sex-matched healthy volunteers served as controls. Plasma samples collected at ICU admission and days 2–4 were analyzed using a novel POC multiplex micro-ELISA platform and compared with established in-house and commercial ELISAs using Spearman rank correlation and receiver operating characteristic (ROC) analysis. Additional complement activation markers (C3bc, C3bBbP, C1s/C1-INH, MASP-1/C1-INH and C4d) were assessed to explore specific pathway involvement.

**Results:**

TCC levels were significantly elevated in patients (median 3575, IQR 2048–6504 mAU/ml) compared with controls (median 1286, IQR 1032–1903 mAU/ml; *p* < 0.0001) at both sampling time points. The POC assay demonstrated strong rank correlation with the in-house TCC ELISA (r = 0.83, *p* < 0.001) and the commercial Hycult TCC ELISA (r = 0.88, *p* < 0.001). The POC TCC assay accurately discriminated patient samples above and below the ELISA-defined threshold (AUC 0.97, *p* < 0.001). In contrast, C3 and C3d showed limited patient-control discriminatory ability and poor POC–ELISA agreement. C3bc and C3bBbP were elevated in patients, suggesting alternative pathway amplification. C1s/C1-INH and C4d were not significantly elevated, whereas MASP-1-C1-INH complexes were decreased in patients compared to healthy controls.

**Conclusion:**

Point-of-care TCC quantification is feasible, correlates well with established ELISAs and accurately identifies elevated TCC levels in critically ill patients. Although formal method-comparison analyses were not possible to establish, TCC emerged as the most robust marker of complement activation in this ICU cohort using the evaluated POC prototype. POC testing may facilitate timely identification of complement activation in acute care settings and support integration of complement-directed diagnostics and therapeutics into clinical workflows.

## 1 Introduction

The complement system is a central component of human innate immunity, rapidly identifying and eliminating pathogens and damaged cells through three activation routes: the classical, lectin, and alternative pathways. Together these pathways converge on the cleavage of plasma proteins C3 and C5 to generate effector mechanisms such as opsonization, anaphylatoxin release, and assembly of the terminal complement complex, also known as TCC or C5b-9 (1–3). Dysregulated complement activation contributes to a wide range of inflammatory and thrombotic diseases, and its clinical relevance is now widely recognized (4).Over the last few decades, complement biology has entered a therapeutic revolution in complement-targeted interventions (5). A growing number of diseases have been identified as potential candidates for complement inhibiting therapy, such as various kidney diseases, autoimmune disorders and microangiopathies of diverse origin (6). Beyond the established C5 inhibition with eculizumab, numerous new inhibitors targeting different parts of the complement system have entered the field (7). To date, fourteen complement-modulating drugs have been approved by the FDA for the treatment of eleven different diseases. Extensive off-label use of complement-inhibiting therapies further underscores the clinical demand for these interventions (8). This expanding therapeutic landscape has intensified the need for timely and accurate assessment of complement activation to identify treatable phenotypes, guide clinical decision-making, and monitor therapeutic responses.

With complement-directed therapies now entering clinical practice, rapid and reliable diagnostics are becoming essential, particularly in clinical emergency settings (9). Although enzyme-linked immunosorbent assays (ELISAs) remain the gold standard for detecting complement activation products, they are time-consuming and laboratory-bound, limiting their utility in situations where early and rapid recognition of complement dysregulation may influence management (10, 11). Point-of-care assays capable of detecting complement activation, especially downstream markers such as TCC, could help narrow this diagnostic gap. Recent studies on multiplex micro-ELISA platforms have shown that rapid multi-analyte quantification with low sample volume requirements and short processing times is feasible, supporting the broader translational potential of such systems beyond conventional laboratory settings (12).

In this study, we evaluated whether a rapid and clinician-operated multiplex micro-ELISA platform for C3, C3d and soluble TCC correlates with conventional laboratory ELISAs. Using samples from a heterogeneous ICU population, we also assessed a complementary set of established activation markers (C3bc, C3bBbP, C1s/C1-INH, MASP-1/C1-INH and C4d) to characterize pathway-specific patterns of complement activation in critical illness. Collectively, this work aims to determine whether point-of-care quantification of TCC, C3 and C3d is feasible and clinically informative, and to explore the potential for integrating rapid complement diagnostics into acute care workflows.

## 2 Materials and methods

### 2.1 Study population

Patients aged ≥18 years presenting with critical illness were prospectively enrolled from a mixed medical and surgical intensive care unit (ICU) at a university hospital. Eligible participants were selected pragmatically from clinical conditions in which complement activation has been reported or is considered biologically plausible based on current literature (8, 9), although not necessarily the primary driver of pathogenesis. Clinical diagnoses included cardiac arrest with return of spontaneous circulation, sepsis, subarachnoid hemorrhage, organ donors by brain death, and microangiopathic conditions such as pre-eclampsia and sickle-cell crisis. To ensure representation of samples with markedly elevated TCC levels, three patients undergoing elective cardiac surgery with cardiopulmonary bypass were included, as cardiopulmonary bypass is a well-known activator of the complement system (13). The principal aim of this exploratory feasibility study was to evaluate assay performance across a broad range of clinical conditions and complement activation levels. Accordingly, the intention was to enroll a heterogeneous cohort, rather than recruit patients based on predefined laboratory criteria or diagnostic thresholds. Routine laboratory parameters were analyzed at the Department of Clinical Biochemistry at Oslo University Hospital. In addition, level of organ-supportive care and length of stay in the ICU were recorded. Fifteen age- and sex-matched healthy volunteers were recruited as negative controls. No formal *a priori* sample size calculation was performed. The sample size was determined pragmatically based on the number of available POC assay test chips during the study period.

### 2.2 Development and analytical validation of the multiplex POC assay

The POC TCC, C3, and C3d multiplex assay was developed by GENSPEED Biotech (Austria) in collaboration with Hycult Biotech (The Netherlands) on a microfluidic chemiluminescent multiplex micro-ELISA platform. TCC, C3, and C3d specific antibodies (Hycult Biotech) were immobilized onto polymer foils using non-contact microarray spotting (sciFLEXARRAYER, SCIENION, Germany). Following spotting and drying, the antibody-coated foil was ultrasonically welded to a microfluidic top layer containing reaction channels and fluidic inlets, forming the biochip. The chip integrates sample and reagent inlets, an antibody microarray reaction zone, and an absorbent reservoir that drives capillary flow, enabling automated analysis of small sample volumes within 22 minutes (12). Assay conditions, including antibody selection, spotting parameters, reagent concentrations, and sample dilution protocols, were optimized for analytical performance. Analyte-specific calibration curves were established using calibrator materials similar to those used in the corresponding singleplex ELISAs. TCC calibrator consisted of zymosan-activated pooled human serum (37 °C, 1 h) terminated with 20 mM EDTA and assigned a value of 1000 AU/ml. C3 calibrator was prepared from pooled human EDTA-plasma calibrated against purified human C3, while C3d calibrator was generated by spiking pooled human EDTA-plasma with purified human C3d (Complement Technology, TX, USA). For analytical validation, plasma samples were diluted 1:10 (3 µl plasma, 27 µl dilution buffer) and incubated for 5 minutes at room temperature, before 25 µl was applied to a microfluidic test chip in the GENSPEED R2 analyzer. Following automated sample processing, chemiluminescent detection was initiated by addition of one drop of detection reagent. All subsequent assay steps, signal acquisition, and data analysis were performed automatically. Analytical validation included assessment of LLoQ, ULoQ, analytical measuring range, signal-to-noise ratio (S/N), dilution linearity, recovery, repeatability (intra-assay variation), and reproducibility (inter-assay variation between operators). Acceptance criteria were R² ≥0.99, CV ≤25% for linearity and precision, recovery of 80-120%, and S/N >10. LLoQ and ULoQ were defined as the lowest and highest calibrator concentrations, respectively, with signals differing by ≥3 SD from blank or the adjacent lower calibrator. Accuracy and precision requirements for multiplex microfluidic POC assays are often less stringent than those for laboratory-based singleplex ELISAs because multiplexing and miniaturization introduce additional sources of analytical variability.

### 2.3 Blood sampling protocol and POC analyses of TCC, C3 and C3d

Peripheral venous blood was obtained from the patients in ethylenediaminetetraacetic acid (EDTA) tubes at admission to the ICU and again after 2–4 days. The cardiopulmonary bypass patients were sampled once during cardiopulmonary bypass and once postoperatively after 3 days. Samples were immediately placed on crushed ice and centrifuged within 30 minutes at 2500 x *g* for 20 minutes at 4 °C, assuring complete halt of complement activation as described previously (14). Plasma was then collected and immediately analyzed for TCC, C3 and C3d using the newly developed POC multiplex micro-ELISA. The total turnaround time from sample preparation to result generation on the POC platform was approximately 45 minutes per sample, including 22 minutes of automated analysis by the GENSPEED R2 analyzer. Following the POC protocol, samples were diluted 1:10 for POC analysis of TCC, C3 and C3d in a sample buffer provided by the manufacturer. Remaining plasma was aliquoted and stored at -70 °C for subsequent ELISA batch analyses.

### 2.4 ELISA protocol for complement biomarkers

Soluble TCC concentrations were quantified using an established in-house sandwich ELISA, in which the mAb aE11 captures the C9 neoepitope exposed after incorporation of C9 into the C5b-9 complex, and using a biotinylated anti-C6 monoclonal antibody for detection, originally described in 1985 (15) and later modified (16). In addition, we assessed TCC using a commercial ELISA kit provided by Hycult Biotech. For the ELISAs, samples were diluted 1:4 for in-house TCC, 1:10 000 for C3 and 1:10 for C3d and Hycult TCC.

Additional complement biomarkers were quantified as follows: C3bc (17) and C3bBbP (18) and were quantified using in-house ELISA assays; C3, C3d, MASP-1/C1-inhibitor complexes and C1s/C1-inhibitor complexes were determined using commercial ELISA kits (Hycult Biotech), and C4d using a commercial ELISA kit (SVAR Life Science, Malmö, Sweden).

### 2.5 Statistical analyses

Continuous variables are presented as medians with interquartile ranges (25^th^–75^th^ percentile), and categorical variables as counts and percentages. Group comparisons were performed using the Wilcoxon signed-rank, Wilcoxon rank-sum (Mann-Whitney U) and Kruskal-Wallis tests. POC assay performance was evaluated by calculating the coefficient of variation (CV%), expressed as the standard deviation divided by the mean x 100% (CV = SD/mean × 100). Admission samples were assessed for group differences between patients and healthy controls. Agreement between the POC TCC assay and the traditional ELISA was assessed using Spearman’s rank correlation coefficient using both admission and follow-up patient samples. This approach was chosen to increase the number of observations and to capture a broader range of TCC concentrations, as complement activation may change substantially during the course of critical illness. Because the primary objective was analytical comparison, each paired measurement was considered an independent observation for method-comparison purposes.

Receiver operating characteristic (ROC) analysis was performed using patient admission samples to assess the diagnostic performance of the POC assay. A TCC level >0.7 CAU/ml measured using the in-house ELISA was pre-defined as a positive threshold for elevated complement activation in ROC analyses (16) and POC-derived TCC was the index variable. The optimal POC TCC cut-off-value was determined by maximizing the Youden index.

Statistical analyses were performed using Excel (Microsoft Corporation, Redmond, WA), Stata (version 18.0, StataCorp, College Station, TX) and GraphPad Prism (version 10.4.1, GraphPad Software, San Diego, CA).

## 3 Results

### 3.1 Study population

**Figure 1:**
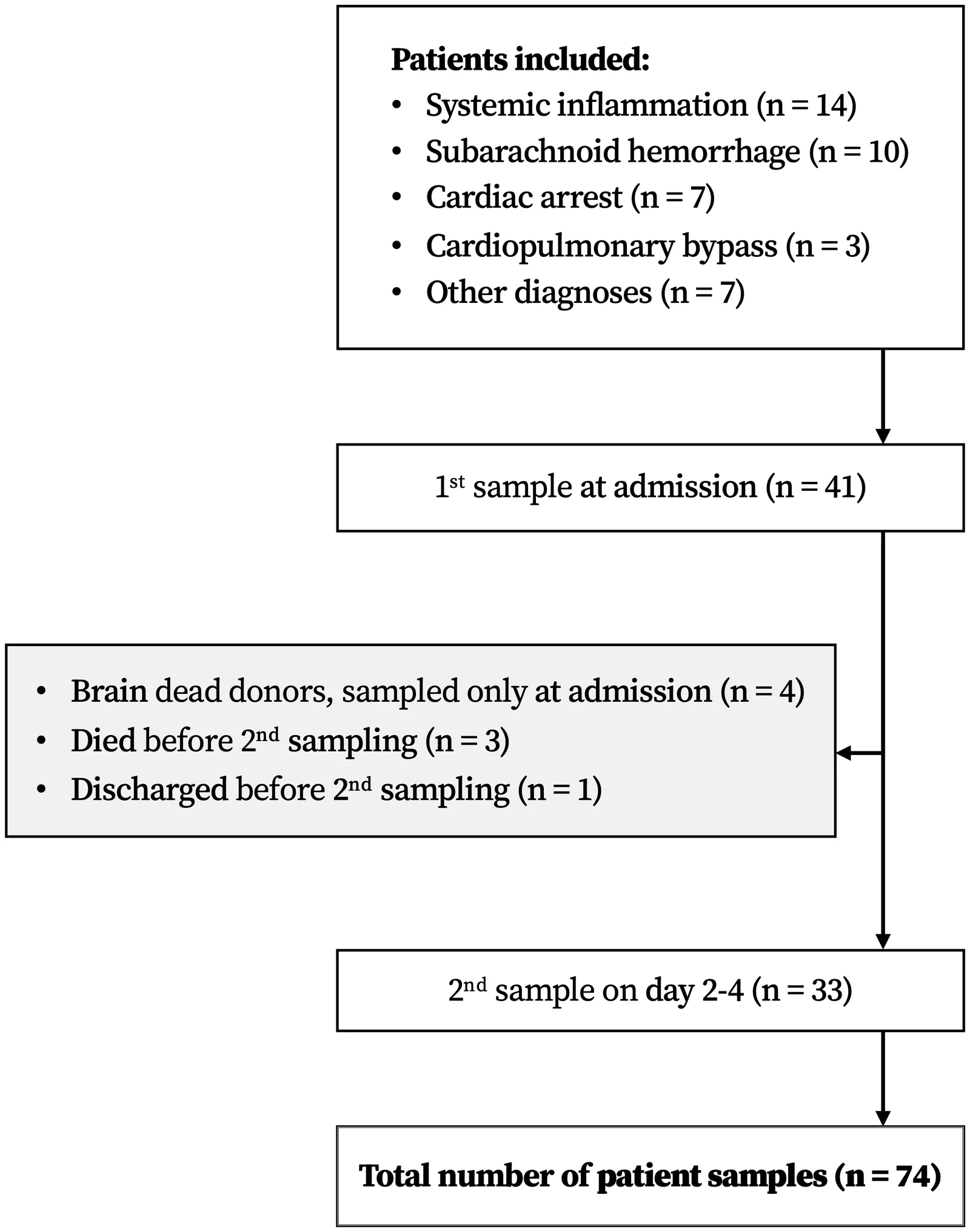
Flow chart of patient population in this study. Enrollment and availability of blood samples at each time-point. A subset of patients contributed only admission samples due to clinical circumstances such as brain death at inclusion, death or discharge prior to second sampling.

The study population comprised 38 critically ill patients and three cardiopulmonary bypass patients with a median age of 54 years, of whom 54% were female. The underlying conditions were severe and heterogeneous, most commonly systemic inflammatory conditions such as sepsis, shock, cardiac arrest with return of spontaneous circulation, subarachnoid hemorrhage and brain death (**Figure 1**). Markers of inflammation and organ dysfunction were elevated with a mean C-reactive protein of 135 mg/l and a mean creatinine of 123 µmol/l. Mortality was 32% during the ICU stay and 39% within 30 days of the first sampling date. Seven patients died before day 2–4 sampling. Study population characteristics are summarized in **Table 1**.

**Table 1:** Patient characteristics during admission to the intensive care unit (ICU)^a^ .

Characteristic	All patients, *n* = 41
Demographics and length of stay	
Age, year (median, IQR)	54 (45–65)
Female sex	22 (53.7%)
Body mass index (median, IQR)	27.9 (23.6–30.7)
ICU length of stay in days (median, IQR)	5 (2–9)
Disease category	
Systemic inflammatory condition	14 (34%)
Subarachnoid hemorrhage incl. donors by brain death	10 (25%)
Cardiac arrest	7 (17%)
Cardiopulmonary bypass (cardiac surgery)	3 (7%)
Other	7 (17%)
Comorbidity	
Hypertension	10 (24.4%)
Diabetes mellitus	5 (12.2%)
Cardiac failure	3 (7.3%)
Kidney failure	2 (4.9%)
Disease severity and supportive care	
C-reactive protein, mg/l (mean, 95% CI)	135 (92.1–177)
Creatinine, µmol/l (mean, 95% CI)	123 (94–153)
Mechanical ventilation	35/41 (85.4%)
Days on invasive mechanical ventilation (median, IQR)	3 (1–7)
Vasoactive support	32/41 (78.0%)
Continuous renal replacement therapy, CRRT	7 (17.1%)
Mortality	
In ICU	13 (31.7%)
30 days after first sampling date	16 (39.0%)

a Values are given as median (interquartile range), mean (95% CI) and number (percent) as indicated.

### 3.2 Analytical performance of the multiplex POC assay

Analyte-specific standard curves for TCC, C3, and C3d were successfully established using calibrator materials with assigned concentrations. Evaluation of the standard curves demonstrated broad quantifiable ranges, with LLoQ–ULoQ values of 300–40,000 mAU/ml for TCC, 3–500 µg/ml for C3, and 20–8,000 ng/ml for C3d. Sample linearity was assessed by calculating the CV% across at least three consecutive sample dilutions. TCC, C3, and C3d demonstrated good dilution linearity, with CV values of 23.4%, 3.7%, and 19.2%, respectively, all meeting the predefined acceptance criterion of ≤25%. Next, assay accuracy and precision were assessed by evaluating recovery, intra- and inter-assay variation. Recovery experiments in EDTA-plasma samples with known analyte concentrations demonstrated recoveries of 82%, 88%, and 98% for TCC, C3, and C3d, respectively. Intra-assay CVs were ≤23% (TCC), ≤18% (C3), and ≤20% (C3d), while inter-assay CVs were ≤20.4%, ≤10.7%, and ≤13.3%, respectively. A summary of all assay performance characteristics is provided in **Table 2**.

**Table 2:** Analytical performance parameters multiplex POC micro-ELISA.

Analytical parameter	TCC (mAU/ml)	C3 (µg/ml)	C3d (ng/ml)
Range (LLoQ/ULoQ)	300-40.000	3-500	20-8000
Linearity of dilutions (CV%)	23.4	3.7	19.2
Recovery (%)	82	88	98
Intra-variation (CV%)	≤23	≤18	≤20
Inter-variation (CV%)	≤20.4	≤10.7	≤13.3
Sample volume (µl)	3	3	3
Assay time (minutes)	22.5	22.5	22.5
Calibrator source	Human serum pool, zymosan activated, set to 1000 AU/ml	Human EDTA plasma pool with known C3 concentration	Human isolated C3d protein spiked in heat-inactivated base matrix

### 3.3 Point-of-care measurements of TCC, C3 and C3d

**Figure 2:**
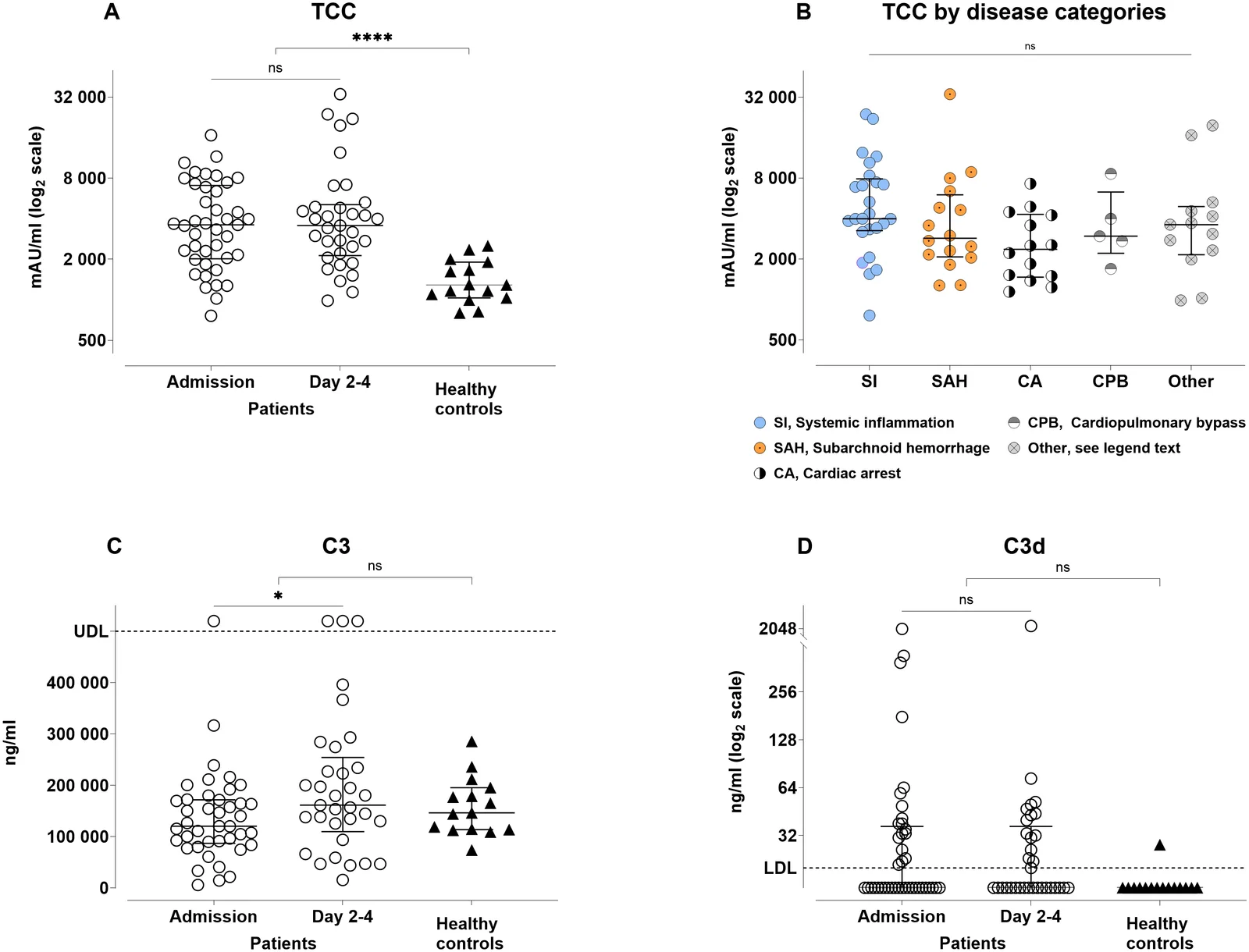
**(A)** TCC measured using the POC platform in patients at admission, on day 2-4, and in healthy controls. **(B)** TCC measured at admission across different disease categories. “Other” reflects diagnoses such as acute respiratory distress syndrome (ARDS), tumor lysis syndrome, pre-eclampsia, sickle-cell crisis and catastrophic anti-phospholipid syndrome. **(C)** and **(D)**: C3 and C3d measured using the POC platform in patients at admission, on day 2-4, and in healthy controls. Data presented as medians and interquartile range. Statistical comparisons performed: Wilcoxon signed-rank test (sampling time points), Mann-Whitney U test (patients versus healthy controls), and Kruskal-Wallis test (disease categories); ****p < 0.0001, *p < 0.05; ns = not significant. UDL, Upper detection limit; LDL, Lower detection limit.

TCC levels measured by the POC assay were significantly higher in patients compared to healthy controls (median 3575, IQR [2048–6504] vs 1286 [1032–1903] mAU/ml; *p* < 0.0001), both at admission (3595 [2013–7064] mAU/ml, *p* < 0.0001) and on day 2–4 (3554 [2130–5081] mAU/ml, *p* < 0.0001) (**Figure 2A**). Despite considerable variability, TCC levels were elevated in patients irrespective of underlying diagnosis (**Figure 2B**).

Quantification of total C3 levels measured by the POC assay did not differ between patients at admission and healthy controls (120–316 [86 852–171 736] vs 146–177 [113 683–195 274] ng/ml, *p* = 0.167) (**Figure 2C**).

C3d measured by the POC assay revealed levels below the detection limit in 68% of all samples (**Figure 2D**). Among detectable values, no difference was observed between patients at admission and healthy controls.

### 3.4 Point-of-care correlations with ELISAs

**Figure 3:**
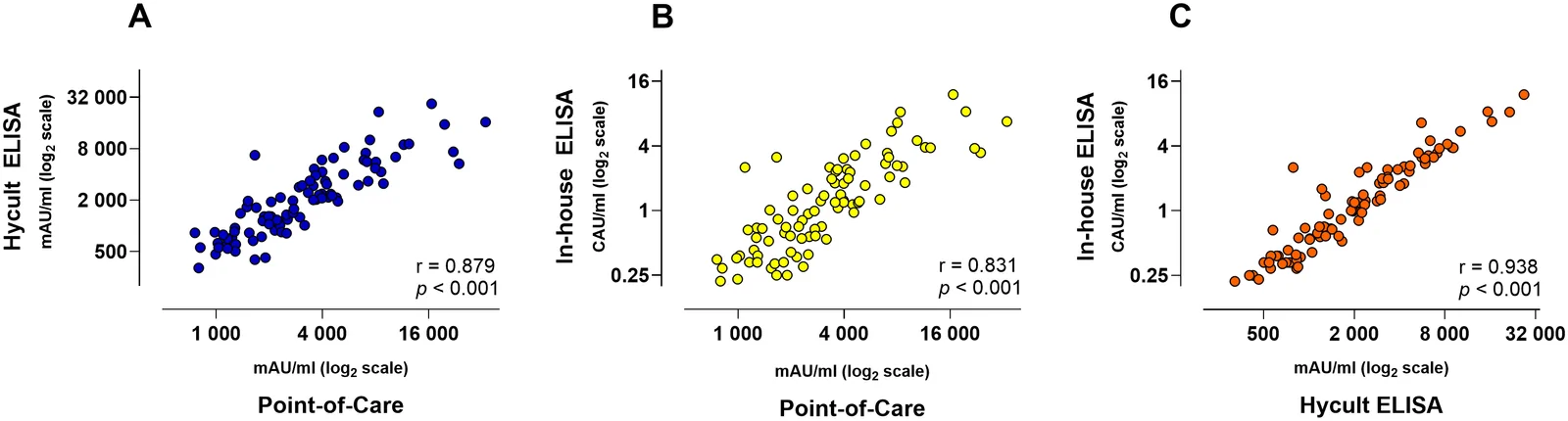
Spearman rank correlations comparing TCC measurements obtained using **(A)** the POC assay and Hycult ELISA; **(B)** the POC assay and in-house ELISA; **(C)** Hycult ELISA and in-house ELISA.

Non-parametric Spearman analyses demonstrated strong positive rank correlations between TCC quantified in all samples by the POC assay and the Hycult ELISA (r = 0.88, 95% CI 0.82–0.92, *p* < 0.001), and by our in-house ELISA (r = 0.83, 95% CI 0.75–0.89, *p* < 0.001) (**Figures 3A, B**). The two ELISA assays demonstrated similar strong correlation (r = 0.94, 95% 0.91–0.96, *p* < 0.001) (**Figure 3C**).

To assess the potential impact of two repeated measurements in patients, all TCC correlation analyses were repeated using only the admission sample from each patient, together with samples from healthy controls (n = 56). The results were consistent with those obtained using the full dataset (**Table 3**).

**Table 3:** TCC Spearman rank correlations according to sample size.

Correlation statistic	POC vs In-house ELISA	POC vs Hycult ELISA	In-house vs Hycult ELISA
All patient and healthy control samples (n = 89)			
Spearman r	0.83	0.88	0.94
95% CI	0.82-0.92	0.75-0.88	0.91-0.96
Patient admission and healthy control samples only (n = 56)			
Spearman r	0.78	0.81	0.89
95% CI	0.65-0.87	0.68-0.88	0.82-0.93

There was no significant correlation between the C3 POC assay and C3 ELISA (r = 0.10, *p* = 0.355). No significant correlation was found between the C3d POC and C3d ELISA results among the 26 values detectable by both methods (r = 0.34, *p =* 0.08).

### 3.5 Receiver operating characteristic of the POC TCC assay

**Figure 4:**
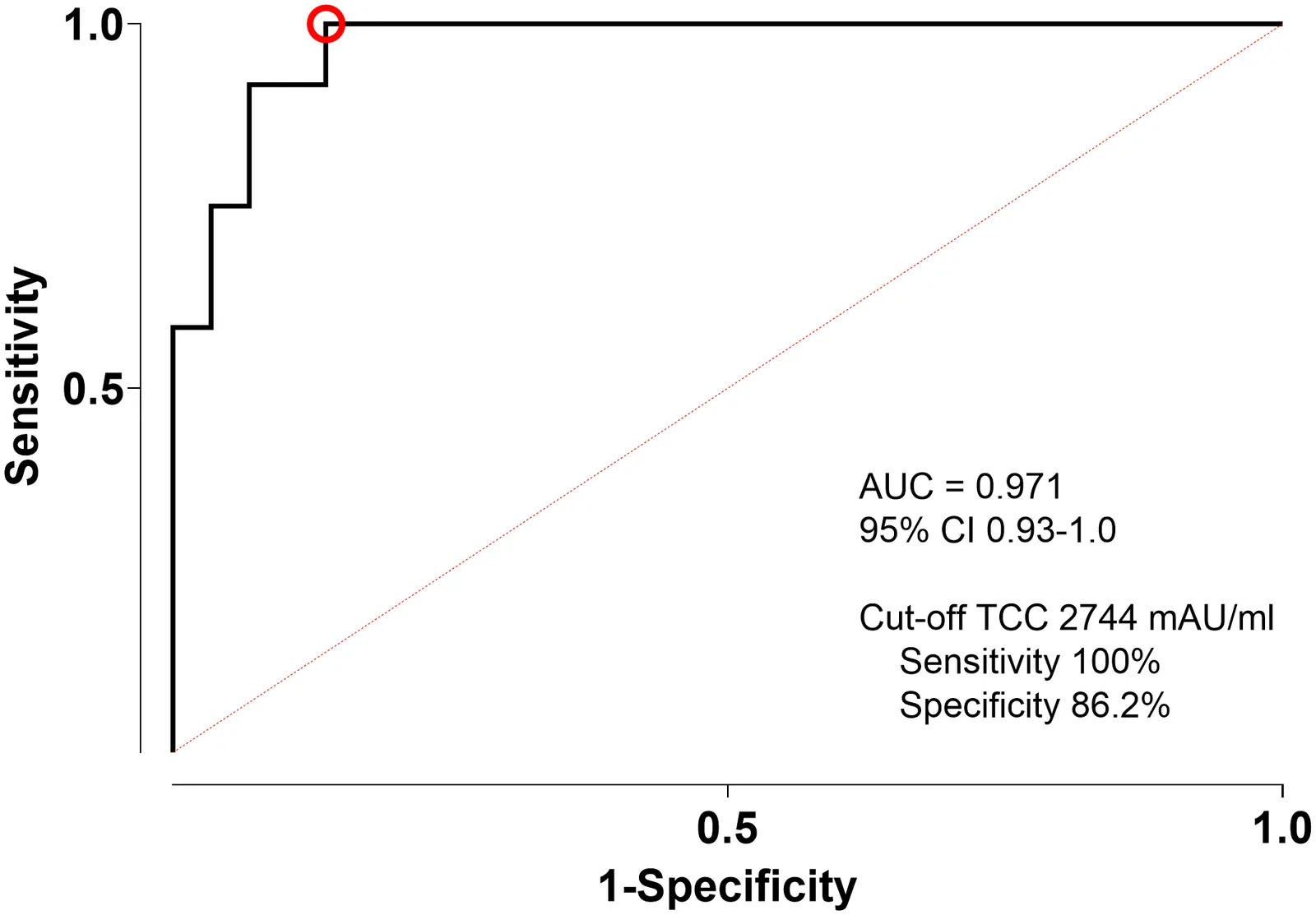
Receiver operating curve of the POC TCC assay for identification of elevated TCC levels (>0.7 CAU/ml in the reference in-house ELISA) in patients at admission. The optimal cut-off value was defined by the maximum Youden index.

To quantify the ability of the POC TCC assay to identify samples with elevated TCC as defined by the in-house ELISA reference, a receiver operating characteristic (ROC) analysis was performed. Patient admission samples with TCC values >0.7 CAU/ml were classified as ELISA-positive (n = 30 in a total of 56 samples). The POC assay demonstrated strong discriminatory performance, with an area under the ROC curve (AUC) of 0.97 (95% CI 0.93-1.0). The optimal POC threshold identified by the maximum Youden index was 2744 mAU/ml, corresponding to a sensitivity of 100% and a specificity of 86.2% (**Figure 4**). All healthy control TCC POC values were below 2744 mAU/ml.

### 3.6 Quantification of upstream complement activation products and activation routes

**Figure 5:**
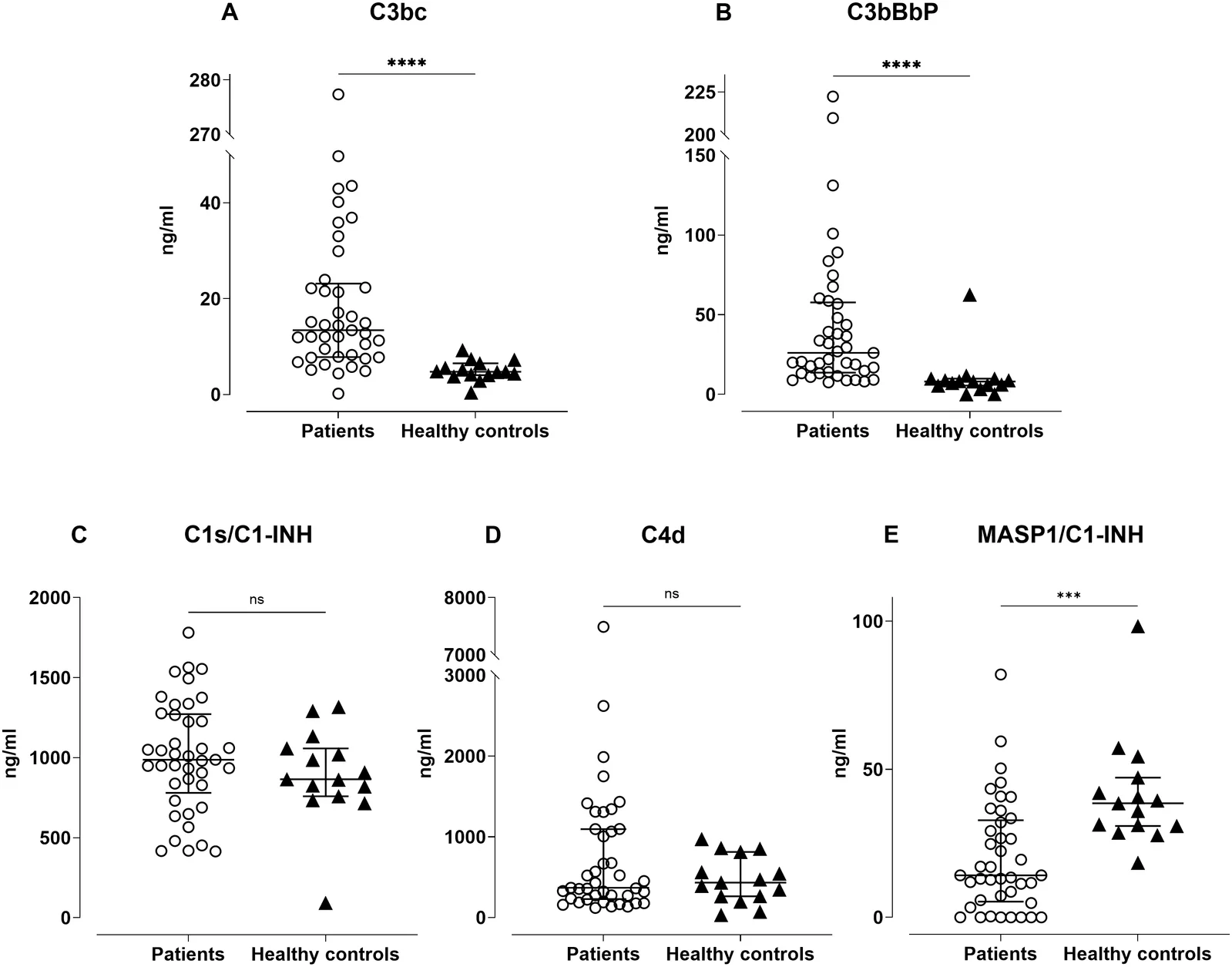
Upstream complement activation markers in patients at admission and in healthy controls. **(A)** C3bc (common pathway); **(B)** C3bBbP (alternative pathway amplification); **(C)** C1s/C1-INH complex (classical pathway), **(D)** C4d (classical and lectin pathway); and **(E)** MASP1/C1-INH complex (lectin pathway). Data are shown as individual values with medians indicated. Mann-Whitney U test; ****p < 0.0001; ***p < 0.001; *p < 0.05, ns, not significant.

C3bc (common pathway) was significantly higher in patients at admission than in healthy controls (median 13.4, IQR [7.8-23.2] vs 4.8 [4.0-6.5] AU/ml; *p* < 0.0001) (**Figure 5A**). C3bBbP (alternative pathway) was similarly elevated in patients compared to controls (26.1 [13.7-57.7] AU/ml vs 8.1 [5.5–9.9] AU/ml; *p* < 0.0001) (**Figure 5B**).

C1s/C1-inhibitor complexes (classical pathway) did not differ between patients at admission and controls (987.0 [779.9-1271] vs 865.1 [758.8–1057] ng/ml; *p* = 0.270) (**Figure 5C**), nor did C4d levels (both classical and lectin pathways; 369.3 [228.3-1095] vs 432.7 [262.3–812.1] ng/ml; *p* = 0.570) (**Figure 5D**).

MASP-1/C1-inhibitor complexes (lectin pathway) were significantly lower in patients at admission compared to controls (14.2 [5.34–32.8] vs 38.52 [30.9–47.2] ng/ml, *p* = 0.0002) (**Figure 5E**).

## 4 Discussion

This study demonstrates that a point-of-care micro-ELISA can detect terminal complement activation in critical illness, with strong Spearman rank correlation to established laboratory ELISAs. ROC analysis found that TCC values obtained with the POC platform discriminated well between samples with and without TCC activation as defined by the reference in-house ELISA. C3 and C3d within the same POC system did not show comparable performance. TCC showed clear separation between patients and healthy controls, elevated levels spanning diverse disease categories, and consistent correlation across analytical platforms. These characteristics support TCC as a potentially relevant biomarker of complement activation in critically ill patients, and therefore a suitable target for POC testing.

The POC TCC assay demonstrated strong rank correlation with both established TCC ELISA methods, comparable to the correlation observed between the two ELISA assays. Correlation analyses were based on Spearman’s rank coefficients, assessing concordance in sample ranking across platforms rather than numerical agreement. Correlation evaluates directional similarity only (i.e., both high or both low within a sample) and does not assess magnitude of differences or systematic bias. Although the assays consistently identified samples with high or low TCC levels, differences in absolute concentrations were observed. This is a well-recognized challenge in complement biomarker assessment, attributable to differences in assay design and calibration. Conventional ELISAs operate under static conditions with long incubation times, whereas the microfluidic POC assay relies on flow-based analyte capture and shorter reaction times, resulting in different assay dynamics (19). In addition, limited assay standardization remains a challenge in the complement field (20, 21). Although similar calibrators were used, neither the POC assay nor the ELISAs are currently calibrated against a universal reference standard, contributing to differences in absolute concentrations between methods. Harmonization of complement assays remains a key challenge within the field and is currently being addressed through standardization initiatives led by the International Complement Society (22, 23). Future alignment of the POC assay with emerging international standards will further improve comparability between methods. Consequently, the assays should not be considered interchangeable, and the present study does not demonstrate method equivalence or support direct assay substitution. Formal assessment of quantitative agreement would require assay standardization and agreement analyses such as Bland-Altman evaluation, which were beyond the scope of the present study (24).

The ROC analysis was performed to assess the ability of the POC TCC assay to identify samples with elevated TCC using the in-house ELISA as the reference method. It demonstrated good discrimination between samples with normal and high TCC levels. This suggests that the TCC POC assay can identify samples with elevated complement activation as defined by the already established ELISA. However, these findings should be interpreted in context of the current state of complement biomarker standardization. There is currently no universally accepted clinical cut-off for elevated TCC, and assays often report arbitrary units calibrated to an internal reference rather than absolute concentrations. The threshold of >0.7 CAU/ml used in the present analysis is based on the previously published upper reference limit among healthy blood donors for this particular assay (16). Accordingly, the derived POC cut-off should be regarded as specific to this analytical framework and requires additional validation prior to broader application. Despite these limitations, the ROC analysis provides additional evidence that the POC assay can correctly classify samples according to ELISA-defined TCC status in an ICU patient cohort.

Unlike TCC, POC testing of C3 and C3d demonstrated limited utility in this cohort. Total C3 concentrations did not differ between patients and healthy controls and demonstrated poor correlation between POC and ELISA. C3d levels were most frequently near or below assay detection limits, with poor correlation between the few sample values detectable by both methods. These findings likely reflect both biological and analytical factors, including normally high plasma concentrations of C3 in healthy subjects (3), accelerated hydrolysis by interactions with a number of biological and artificial surfaces (25) and the rapid turnover and low circulating concentrations of C3d (26). Analytically, the POC assay is challenged by substantial differences in plasma concentrations among the three analytes, which are measured using a single dilution. Our data indicate that the chosen sample dilution and reagent amounts are optimal only for TCC detection.

The additional analyses of upstream complement markers provide context for the observed terminal pathway activation and help interpret the strong performance of TCC compared with C3 and C3d in the POC system. Increased levels of C3bc and C3bBbP support the interpretation that elevated TCC reflects genuine upstream complement activation through alternative pathway amplification and activation of the central complement cascade, consistent with systemic inflammation and multi-organ failure commonly encountered in the ICU (9, 27–29). This aligns with the established role of the alternative pathway amplification loop, which accounts for nearly 80% of the downstream complement effector activity initially induced by the classical and lectin pathways (30, 31).

In contrast, C1s/C1-inhibitor complexes and C4d did not differ between patients and controls, suggesting that TCC elevation in this ICU cohort was not primarily driven by the classical pathway, or that regulatory mechanisms remain sufficient to prevent measurable accumulation of these markers.

Interestingly, MASP-1/C1-inhibitor complexes were consistently and significantly reduced in patients despite increased TCC levels. This was the only activation product that decreased under conditions of marked complement activation. The significance of this finding is unclear and cannot be determined from the present study. Reduced levels may reflect altered pathway activity, depletion of MASP-1, or other changes associated with critical illness. Given the heterogeneous patient population and absence of additional pathway-specific data, these observations should be interpreted cautiously and require confirmation in future studies.

Interpretation of complement measurements should consider that reduced concentrations of complement proteins may reflect inherited deficiency or acquired consumption secondary to ongoing activation. Concentration measurements alone cannot distinguish these possibilities and should therefore be interpreted together with activation markers such as TCC and the clinical context.

The pathway-specific observations are all exploratory in a small material and should be interpreted with caution. However, they are consistent with the concept that complement activation in critical illness is multifactorial but converges at the level of C3, with downstream activation through C5 culminating in TCC formation (32). Individual upstream markers may show more variable pathophysiological patterns than TCC and are therefore less straightforward to translate into a POC format suitable for ICU and emergency settings. Measurement of TCC provides an integrated readout of complement activation independent of the initiating pathway.

TCC levels were elevated in this mixed and diverse cohort of intensive care unit patients across several broad disease categories. In clinical practice, any therapeutic intervention targeting complement activation would firstly require a clear clinical indication and evidence that complement activation is a key driver of the underlying disease process, rather than merely a marker of illness severity. As such, the POC assay may provide a practical tool to facilitate timely identification of critically ill patients with elevated TCC levels for further evaluation of complement-mediated disease.

Conventional ELISA-based complement testing requires laboratory infrastructure, is labor-intensive and time-consuming, and therefore not suitable for real-time ICU decision-making (33). While the POC platform does not meet the strictest definition of bedside whole-blood testing, it is designed to bring complement analysis closer to the patient than conventional ELISA methods. The current workflow requires plasma processing and is therefore best suited to well-equipped tertiary care or university hospitals. The platform provides results within 45 minutes from sampling and can be operated by clinical personnel in the ICU following brief training, making it particularly suited for acute care settings where timely results may inform therapeutic decision-making.

The POC assay should not be considered as a direct analytical replacement for ELISA, but rather a potential complementary tool for near-patient assessment of patients eligible for complement-targeted therapies. Several factors limit the interpretation of the present findings, including the exploratory study design and relatively small, heterogeneous ICU cohort, the lack of assay harmonization across platforms, including differences in assay design and calibration, and the absence of a universally accepted clinical threshold for elevated TCC levels. Future studies using standardized reference materials and clinically meaningful definitions of complement activation are needed to confirm the diagnostic utility of point-of-care TCC measurements, while quantitative comparability must be assessed using uniformly calibrated assays and formal method-comparison analyses. However, in the context of acute care, timely identification of patients with high complement activation may be of greater clinical value than exact numerical quantification. As complement-targeted therapies expand beyond rare complement disorders into sepsis, trauma, transplantation, neurological disease and other acute inflammatory conditions, rapid complement analysis may become increasingly relevant.

In conclusion, complement activation was common in this heterogeneous but severely ill ICU cohort and was most clearly reflected by terminal complement complex formation. Soluble TCC showed strong discrimination between patients and healthy controls, whereas C3 and C3d analyzed within the same POC assay did not. The evaluated POC TCC assay demonstrated strong correlation with established ELISA methods and excellent discrimination of patient samples with elevated TCC levels according to the reference in-house ELISA. However, differences regarding assay calibration precluded formal method-comparison analyses, and numerical agreement between the POC assay and ELISA could therefore not be established. ELISA remains essential for detailed quantitative analyses, whereas the POC platform may provide a pragmatic approach for timely identification and monitoring of complement activation in critically ill patients.

## Data Availability

The datasets presented in this study can be found in online repositories. The names of the repository/repositories and accession numbers can be found below: https://figshare.com/, https://doi.org/10.6084/m9.figshare.32055606.
