# Correction: Point-of-care assessment of complement activation in the ICU: a feasibility study of rapid terminal complement complex detection in critical illness

**DOI:** 10.3389/fimmu.2026.1984508

**Published:** 2026-09-15

**Authors:** Tam Cung, Søren Erik Pischke, Camilla Schjalm, Bettina Mülleder, Loek Willems, Max Sonnleitner, Erik J.M. Toonen, Tom Eirik Mollnes, Andreas Barratt-Due

**Affiliations:** 1 Department of Immunology, Oslo University Hospital and University of Oslo, Oslo, Norway; 2 Department of Research and Development, Division of Emergencies and Critical Care, Oslo University Hospital, Oslo, Norway; 3 Department of Anesthesia and Intensive Care Medicine, Division of Emergencies and Critical Care, Oslo University Hospital, Oslo, Norway; 4 Institute of Clinical Medicine, Faculty of Medicine, University of Oslo, Oslo, Norway; 5 GENSPEED Biotech GmbH, Rainbach im Mühlkreis, Austria; 6 Research and Development Department, Hycult Biotechnology, Uden, Netherlands; 7 Research Laboratory, Nordland Hospital Trust, Bodø, Norway

**Keywords:** C3, C3d, critical illness, point-of-care measurement, TCC, terminal complement complex, intensive care, complement system activation

Affiliation Institute of Clinical Medicine, Faculty of Medicine, University of Oslo, Oslo, Norway was omitted from the paper. This affiliation has now been added for author(s) Tam Cung, Søren Erik Pischke, Tom Eirik Mollnes and Andreas Barratt-Due.

The original version of this article has been updated.

