# Supplementary material for: Point-of-care assessment of complement activation in the ICU: a feasibility study of rapid terminal complement complex detection in critical illness
Source: Front Immunol. 2026 Aug 11;17:1860988. doi: 10.3389/fimmu.2026.1860988 (PMC13503546; doi:10.3389/fimmu.2026.1860988)
Supplement: Supplementary file 1 [file DataSheet1.pdf]

Supplementary Data S1: AI Prompt Log

Point-of-care assessment of complement activation in the ICU

Cung et al.

Use of Generative AI

During the preparation of this manuscript, the authors used ChatGPT (GPT-5.3; OpenAI) to assist with language editing, rephrasing, and improving clarity of selected text passages. The tool was used to refine wording, adjust tone, and enhance readability, including minor revisions to methodological descriptions. All outputs were critically reviewed, revised, and verified by the authors, who take full responsibility for the final content.

AI-generated outputs were subsequently edited and refined by the authors; therefore, some outputs are not reproduced verbatim in the final manuscript but informed intermediate wording and phrasing.

<u>Prompt Log</u>

Prompt 1 (initial)

Could you rephrase this paragraph to improve flow and readability, and exchange the phrase "we hypothesized" to something more neutral sounding?

"*Rapid identification of complement activation in critically ill patients remains a diagnostic challenge, hindering the prompt recognition and management of complement-driven diseases. The growing use of complement inhibitors underscores the urgent need for rapid, reliable tools to detect complement activation and guide early treatment. Accordingly, we hypothesized that rapid point-of-care quantification of soluble C5b-9 could match the diagnostic performance of conventional ELISAs, and we tested this by sampling ICU patients and directly comparing TCC values measured using a novel Hycult point-of-care platform with those obtained by ELISA.*"

Output

"*Rapid identification of complement activation in critically ill patients remains a diagnostic challenge, limiting timely recognition and management of complement-driven conditions. As the clinical use of complement inhibitors expands, the need for fast and reliable tools to detect complement activation and support early therapeutic decisions has become increasingly evident. In this context, we proposed that point-of-care quantification of soluble C5b-9 might provide diagnostic performance comparable to conventional ELISAs, a premise we evaluated by sampling ICU patients and directly comparing TCC measurements obtained with a novel Hycult point-of-care platform to those generated by standard ELISA.*"

Prompt 2 (initial)

Can you re-phrase to avoid "made up of", instead replace with something more professional sounding: "*Made up of roughly 50 interacting proteins, the complement system can be activated via three distinct pathways*"

Output

"*The complement system, comprising approximately 50 interacting proteins, can be activated through three distinct pathways*"

Prompt 3 (initial)

Can you rephrase this to sound less grand and pompous? "*Collectively, this work aims to determine whether bedside quantification of sC5b-9 is feasible, reliable, and clinically informative, and to lay the groundwork for integrating rapid complement diagnostics into acute care workflows.*"

Output

"*Overall, this study evaluates whether bedside quantification of sC5b-9 is feasible, reliable, and clinically informative, and whether it may contribute to the integration of rapid complement diagnostics into acute care workflows.*"

Prompt 4 (initial)

Rephrase methodological description of ELISA protocol including antibody details.

Output

"*Soluble TCC (sTCC) concentrations were quantified using an established in-house sandwich ELISA. Briefly, a monoclonal antibody recognizing a neoepitope on C9 exposed upon incorporation into the C5b-9 complex was used for capture, and a biotinylated monoclonal antibody targeting C6 served as the detection antibody.*"

Prompt 5 (initial)

Refine sentence:
"*Despite heterogeneity in underlying diagnoses, convergence at the level of TCC was a consistent feature, making the TCC PoC to ELISA correlation especially interesting.*"

Output

"*Despite heterogeneity in underlying diagnoses, convergence at the level of TCC was consistently observed, highlighting the relevance of the correlation between PoC and ELISA measurements.***"**

Prompt 6

Rephrase and shorten sentence on complement pathways and amplification**.**

Output

“*Although all pathways converge on terminal pathway activation, the majority of downstream effector activity is driven by alternative pathway amplification.***”**

Prompt 7 (initial)

Can you help me re-phrase this paragraph to avoid the use of "demonstrated" thrice? "*In conclusion, we have demonstrated that complement activation was common in this heterogeneous ICU cohort and is most clearly reflected by terminal complement complex formation. Soluble TCC demonstrated the strongest discrimination between patients and healthy controls and correlated consistently across analytical platforms. The evaluated PoC sTCC assay demonstrated good agreement with established ELISA methods and may provide a feasible approach for rapid bedside assessment of complement activation.*"

Output

“*In conclusion, complement activation was common in this heterogeneous ICU cohort and was most clearly reflected by terminal complement complex formation. Soluble TCC showed the strongest discrimination between patients and healthy controls and correlated consistently across analytical platforms. The evaluated PoC sTCC assay showed good agreement with established ELISA methods and may provide a feasible approach for rapid bedside assessment of complement activation.”*

Prompt 8 (initial)

Is this formulated correctly? "*Non-parametric Spearman correlation in logarithmic distribution plots between sTCC measurements obtained using (A) the point-of-care assay and Hycult ELISA; (B) the point-of-care assay and in-house ELISA; (C) Hycult ELISA and in-house ELISA.*" I mean to describe Spearman correlation plots shown in graphs with natural logarithmic scales on the Y- and X-axes.

Output

“Spearman correlation analyses of sTCC measurements obtained using (A) the point-of-care assay and Hycult ELISA; (B) the point-of-care assay and in-house ELISA; and (C) Hycult ELISA and in-house ELISA. Data are presented on logarithmic scales on both axes.”
